# Supplementary figures and images for: Analysis of allelic cross-reactivity of monoclonal IgG antibodies by a multiplexed reverse FluoroSpot assay
Source: eLife. 2022 Jul 15;11:e79245. doi: 10.7554/eLife.79245 (PMC9286747; doi:10.7554/eLife.79245)

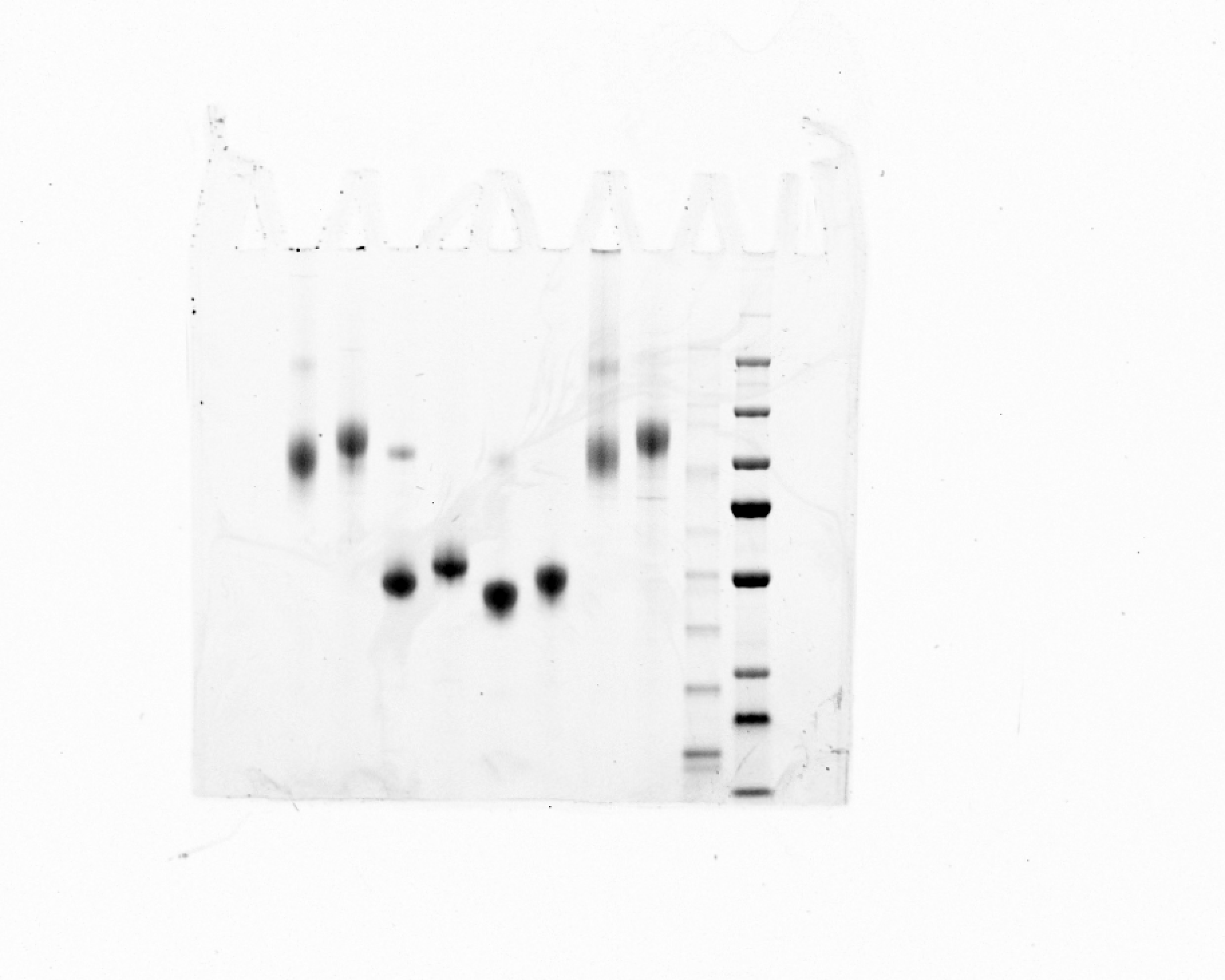

Supplement: Figure 1—figure supplement 1—source data 2. [file elife-79245-fig1-figsupp1-data2.tif]

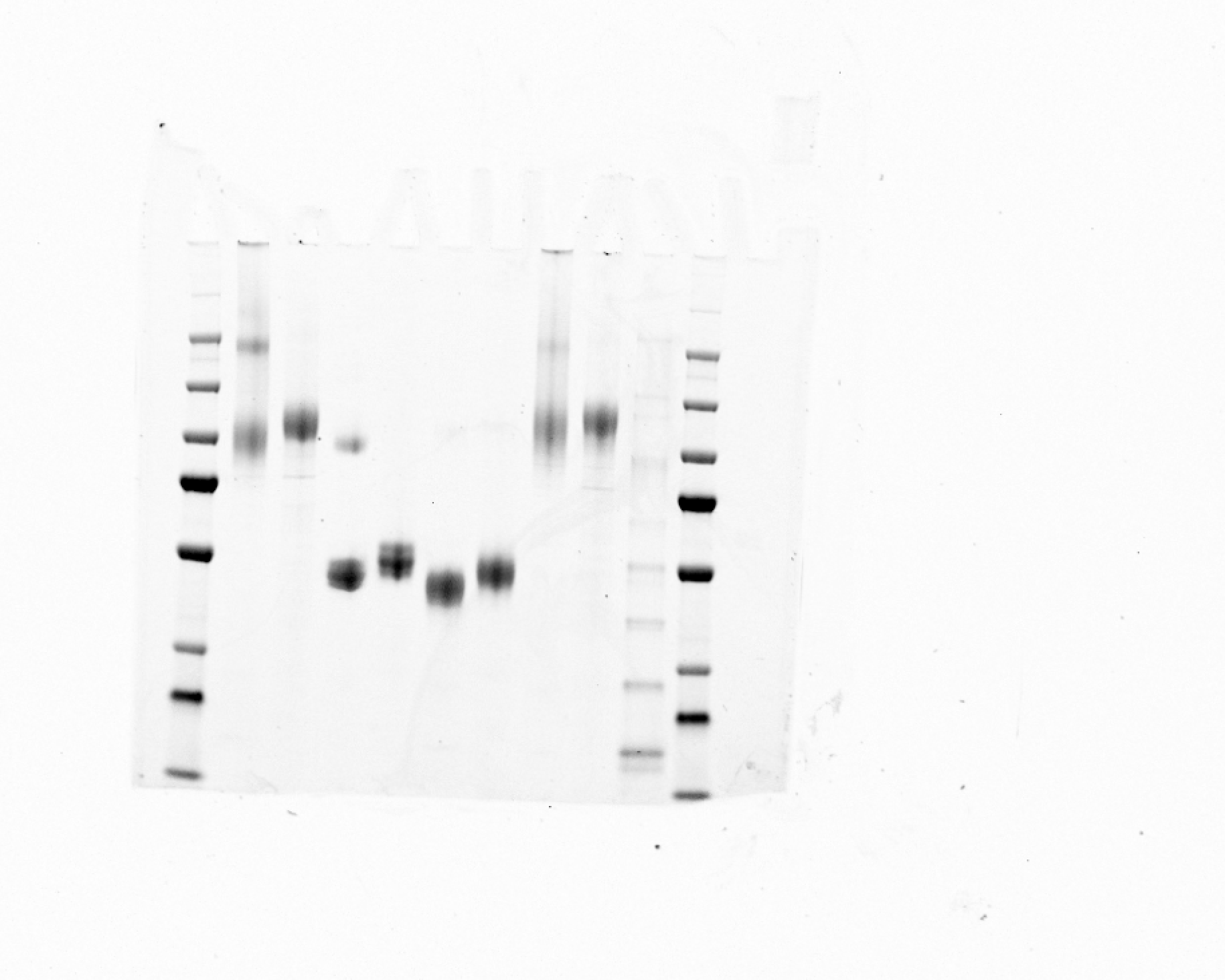

Supplement: Figure 1—figure supplement 1—source data 3. [file elife-79245-fig1-figsupp1-data3.tif]

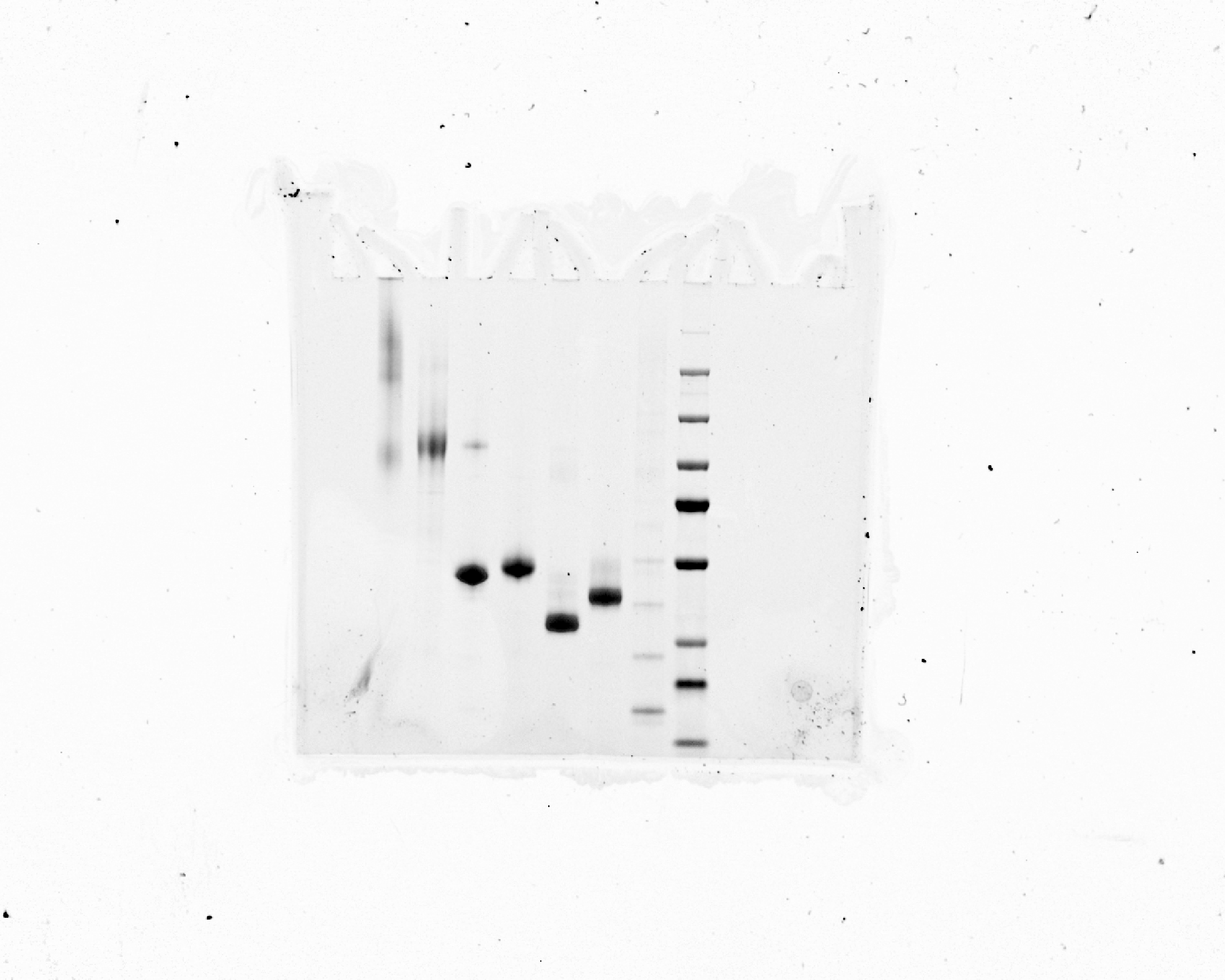

Supplement: Figure 1—figure supplement 1—source data 4. [file elife-79245-fig1-figsupp1-data4.tif]

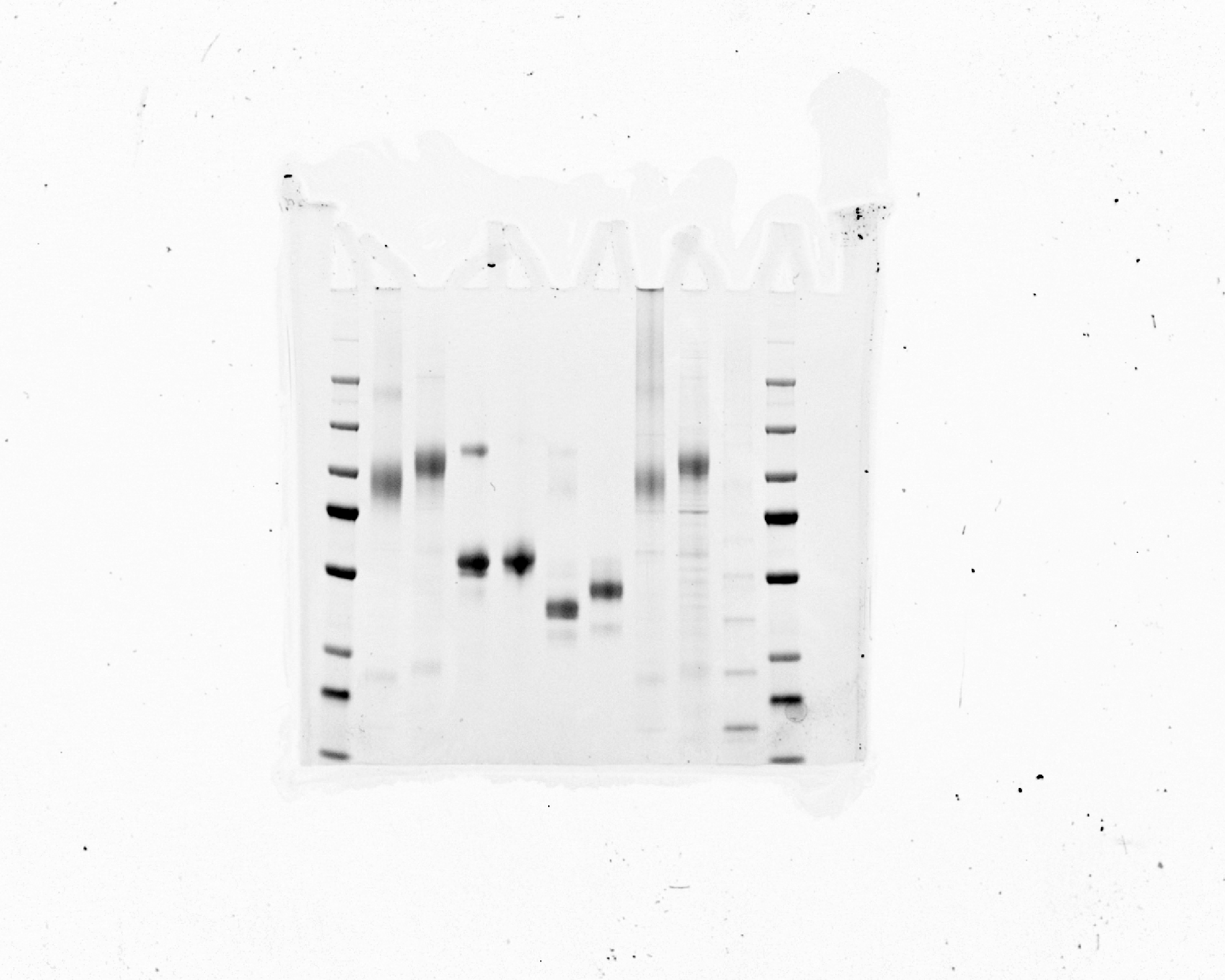

Supplement: Figure 1—figure supplement 1—source data 5. [file elife-79245-fig1-figsupp1-data5.tif]
